# The current state of cataract surgery training in the independent sector

**DOI:** 10.1038/s41433-023-02608-7

**Published:** 2023-06-12

**Authors:** The Ophthalmologists Training Group, Sunil Mamtora, Hasan Naveed, Jeffry Hogg, Zain Juma, Syed Ahmed, Sophie Cowen, Jay Richardson, Samuel Simpson, Adham Youssef, Sohaib Rufai, Guy Hunter, Shahanaz Ahmed, Zhihang Cheng, Adonis El Salloukh, Toby Al-Mugheiry, Mary Henry, Vishal Shah, Kenneth Gilmour, Matthew George, Frederick Burgess

**Affiliations:** The Royal College of Ophthalmologists, 18 Stephenson Way, London, NW1 2HD UK

**Keywords:** Health occupations, Technology

## Introduction

The proportion of cataract surgery performed by the independent sector (IS) has increased dramatically since 2016. A recent RCOphth publication noted significant variation regionally in the provision of cataract surgery by IS providers [1]. Training opportunities in cataract surgery have been a cause for concern by Ophthalmologists in Training nationally in recent years [2]. The pandemic resulted in a hiatus in cataract surgery training for most trainees. Since this time, training opportunities specific to cataract surgery continue to be a serious cause for concern whilst the proportion of cataract operations performed in the IS has increased from 34% in December 2020 to 59% in February 2021 [1].

As a point of contact for Ophthalmologists in Training, the Ophthalmologists Training Group (OTG), a committee of the Royal College of Ophthalmologists receives and reviews concerns from trainees across the four nations and advocates for the high-quality training, including in cataract surgery. There has been a significant body of work undertaken to establish a trainee presence in the IS to ensure the delivery of cataract surgery training following the pandemic [3]. In this article, we provide an overview of the training opportunities currently being made available to Ophthalmologists in Training in the United Kingdom.

## Methods

A data collection exercise was undertaken by the OTG in November 2022 to identify the extent to which training was occurring within the IS on a regional level and the individual experiences of this. The OTG consists of 19 regional representatives who engaged with the trainees they represent in 18 regions. The questionnaire used to gather data can be viewed in Appendix 1.

Data was successfully collected from all 18 regions in the United Kingdom which was reviewed, collated, and analysed by the OTG. A thematic analysis was undertaken using the free-text responses. Data familiarisation was achieved by reading and re-reading the responses, followed by open coding. Codes were contrasted or combined to produce themes. Finally, the themes were reviewed to ensure all codes were accounted for.

## Results

The data collection exercise highlighted that independent sector providers are operating in all 18 regions of the United Kingdom. Seven out of eighteen regions reported trainees being able to access the IS for cataract surgery training opportunities. No junior ophthalmologists in training, defined as trainees within their first 2 years of training, were given access to training opportunities within the IS. Trainees who had an opportunity to access training opportunities in the IS were asked to what extent they agreed with the statement ‘cataract surgery training in the IS, the experience has been positive overall.’ Eighty-five percent of trainees either strongly agreed or agreed.

Thematic analysis was performed for all free-text data, identifying six major themes: limited case complexity, limited case numbers, suboptimal training for junior trainees, administrative burden, complications, and some positive training experiences. Table 1 displays the six major themes and sample responses.

**Table 1:** Thematic analysis of responses in the six identified areas.

Theme	Sample responses
Limited case complexity	“IS has been cherry picking cases with DSD score 1, and leaving more complex cases for NHS trust.” “All straightforward cases on high-volume lists.”
Limited case numbers	“Infrequent lists or trainees only allowed a limited number per list.” “Two separate sites, one offering up to eight cases per list, another offering no more than two per list (as the remaining eight are private patients).”
Suboptimal training for juniors trainees	“No teaching microscopes so juniors trainees weren’t allowed to operate.” “There is an unspoken time pressure as it’s a high volume list, but no trainee has been told to hurry up.” “No opportunity to learn to train others.”
Administrative burden	“Significant paperwork burdens and administrative inefficiencies.” “Commuting distance and difficulties managing timetables.” “The IS provider asked me for a huge amount of paperwork, but not until I got there! Turned up for an all-day list and wasn’t allowed to operate at all that day.”
Complications	“Often complications were handled at the local NHS trust.” “Good experiences managing complications in our deanery with very good plan for post-op follow up. The trainees had the opportunity to follow up their own patient where appropriate with consultant input where needed, otherwise the consultant followed up one of the patients with a complication at the IS provider.”
Positive training experiences	“Many of the members of the wider theatre team are known to trainees from their NHS department, so it is a friendly and familiar environment. The phaco machine and other equipment were largely the same.” “Really gave me confidence as I had low numbers and little opportunity to do more theatres.” “Good experience overall, would be even better with more cases.”

## Discussion

Given the trend of an increasing proportion of cataract operations being performed in the IS, training in the independent sector is essential to be able to provide a sufficiently trained workforce of the future. Several barriers related to trainees being able to access IS training such as indemnity and educational governance related issues, which were previously reported as ‘sticking points’ by the IS have since been overcome [4]. Despite this, we can clearly see that a minority of trainees in a minority of regions are currently able to access cataract surgery training the IS; an issue that remains one of the most concerning issues for Ophthalmologists in Training. Although it has been reported that Ophthalmologists in training have benefited from access to the IS, the reality is that the current training provision in the IS does not come close to being sustainable with regards to training our future workforce [5].

Recent data published by the GMC correlated findings from our data collection exercise [6]. Ophthalmologists in training are expected to have completed 50 full cataract surgeries by the end of their second year of training, the most recent data from the GMC found that only 65% had managed to do so, a significant decrease from previous years. Three-quarters of trainees reported that they needed to access the IS for training opportunities.

The blueprint for delivering cataract surgery in the IS which was recently published by the Royal College of Ophthalmologists following consultation with NHS England, Ophthalmologists in Training and IS providers sets out a minimum expectation of IS providers to deliver training. This will require all IS providers to deliver training on at least 11% of whole cataract surgery operations within two years [7].

## Conclusions

Whilst cataract surgery training in the IS is occurring, opportunities are very limited. In the aftermath of the pandemic, more ophthalmologists in training are struggling to progress with many finding it difficult to undertake the required number of cataract procedures. A big part of this challenge in England is that there has been a huge increase in the proportion of NHS cataract procedures delivered in the IS, without the accompanying increase in training opportunities.

We have found that where training opportunities exist, they have overall been received positively. The blueprint sets out a minimum standard of training delivery required by the IS. The extent to which this is delivered on must be reviewed and enforced as necessary. The OTG will play a key role in reporting and communicating the extent to which Ophthalmologists in Training are able to access cataract surgery training opportunities in the IS.

### Supplementary information

Supplementary information
