# Supplementary material for: The current state of cataract surgery training in the independent sector
Source: Eye (Lond). 2023 Jun 12;37(18):3714–5. doi: 10.1038/s41433-023-02608-7 (PMC10697933; doi:10.1038/s41433-023-02608-7)
Supplement: Supplementary file 1 — Supplementary information [file 41433_2023_2608_MOESM1_ESM.pdf]

Cataract Surgery Training in the Independent Sector

OTG Data Collection Exercise 2022

* Indicates required question

1. Which deanery are you representing? *

Mark only one oval.

- Severn
- South Thames (KSS)
- Academic
- North Thames
- Peninsula
- North West
- Northern
- Northern Ireland
- East Midlands
- Wessex
- Oxford
- Mersey
- Wales
- East of England
- West Midlands
- South Thames
- Scotland West
- Yorkshire
- Scotland East

2. Within your deanery, which independent sector providers are operating and where? *

3. Have trainees in your deanery been on placement with an independent sector provider for cataract surgery training?

Mark only one oval.

- Yes *Skip to question 4*
- No *Skip to question 14*

Deaneries in which trainees have placement within the independent sector for cataract surgery training

4. Which independent sector provider(s) have trainees been placed within, if any? *

5. What grade are the trainees placed within the independent sector? Please tick all that apply

Check all that apply.

- [ ] ST1
- [ ] ST2
- [ ] ST3
- [ ] ST4
- [ ] ST5
- [ ] ST6
- [ ] ST7
- [ ] None of the above
- [ ] Other: ____________________

6. Please specify how many trainees of each grade if more than 1. If you selected 'other' for th previous questions, please specify (e.g. LAT, SAS doctor).

____

7. Please comment on their experience of: a) theatre list frequency; b) case complexity; c) opportunities to operate (e.g. average numbers per list); d) teaching quality. e) opportunities for simulation f) who provides supervision and level of supervision provided. Please provide an overview of the experiences from different providers/trainees.

____
____
____
____
____

8. Please comment on any discrepancies between the experiences of junior trainees (ST1-3) and senior trainees (ST4-7), if applicable.

9. Have any trainees had an intraoperative complication during the surgery? What was their experience, including level of supervision for management, where post-operative follow up took place and who reviewed, access to notes?

10. To what extent do you agree with the following statement? "For trainees in this region undergoing cataract surgery training in the independent sector, the experience has been positive overall."

Mark only one oval.

- Strongly agree
- Agree
- Neither agree nor disagree
- Disagree
- Strongly disagree

11. Please share specific comments that trainees have made in relation to cataract surgery training in the independent sector.

12. Where trainees have been placed in the independent sector to receive training in cataract surgery, has there been an expectation that they also undertake clinics e.g. pre-assessment clinics to make up for the theatre time they are getting - please provide details as appropriate.

13. What do you understand to be the barriers to trainees accessing / accessing further independent sector placements - if possible it might be useful to discuss with your ES/CS/TPD/relevant person.

Skip to question 16

Deaneries in which trainees do NOT have a placement within the independent sector for cataract surgery training

14. Do you have any other comments on this topic? Please share any other comments/feedback that trainees have made about training in the independent sector - especially if you do not have trainees placed in the independent sector within your deaner and have not completed section 2.

15. What do you understand to be the barriers to trainees accessing / accessing further independent sector placements - if possible it might be useful to discuss with your ES/CS/TPD/relevant person.

Closing comments

16. Do you have any other comments on this topic?

This content is neither created nor endorsed by Google.

Google Forms
